# Design of Olanzapine/Lutrol Solid Dispersions of Improved Stability and Performances

**DOI:** 10.3390/pharmaceutics5040570

**Published:** 2013-10-25

**Authors:** Cristina Cavallari, Adamo Fini, Giancarlo Ceschel

**Affiliations:** 1 Department FABIT, University of Bologna, Via San Donato 15, 40127 Bologna, Italy; E-Mail: cristina.cavallari@unibo.it; 2 Montefarmaco OTC S.p.A., Bollate-Milan 20021, Italy; E-Mail: info@montefarmaco.it

**Keywords:** olanzapine, solid dispersion, Lutrol^®^ F68 and Lutrol^®^ F127, Gelucire^®^ 44/14, DSC, HSM, XRD, stability, accelerated release

## Abstract

Eleven solid dispersions containing olanzapine, with carriers of different composition (Lutrol^®^ F68, Lutrol^®^ F127, Gelucire^®^ 44/14), were prepared and examined by thermal (differential scanning calorimetry (DSC); thermomicroscopy (HSM)) and X-ray diffraction (XRD) analysis, both as fresh or aged (one year) samples. Drug and carriers were preliminarily selected in order to avoid problems related to the aging of the formulation, according to the solubility parameters of carriers and drug. These parameters make it possible to predict the low solubility of olanzapine in the carriers (alone or in mixtures). Systems containing only Lutrol (also in the presence of Transcutol^®^) contain the drug in the form of particles of reduced size and in a crystalline form. Gelucire^®^ 44/14 apparently increases the amount of olanzapine dissolved in the solid carrier, but this is presumed to be a metastable state, probably related to the heterogeneous nature of the carrier that delays crystallization of the drug. The high hydrophilicity of the carriers proves suitable to an accelerated and quick release of the drug regardless of aging. Differences in the release profiles between Lutrol- and Gelucire-containing systems were interpreted in terms of the formation of polymer micelles by the Lutrols when in aqueous solution.

## 1. Introduction

Solid dispersions represent formulations for the management (improvement or delay) of the dispersed active agent: their advantages as well as disadvantages have been widely recognized, but solid dispersions do not occupy a notable part of the pharmaceutical market, for the reasons clearly examined in an interesting paper published a few years ago [1]. However, both the knowledge of the chemical and physical behaviour of solid dispersions and of the new materials prepared by the pharmaceutical industry could enable the design of systems where advantages largely overcome disadvantages and make these drug/carrier associations more suitable for practical purposes.

The aim of the present paper was therefore the preparation of stable solid dispersions starting from a critical selection of the materials to be employed, based on the solubility parameters; the stability of the systems, thus obtained, was evaluated by thermal analysis.

A model drug, suitable for a solid dispersion with hydrophilic carriers, should be poorly soluble in water, but also thermally stable and resistant to heating, when the melting method is used to prepare the dispersion; it should be appropriate to be accompanied by a large amount of carrier, in other words, it should commonly have a low dosage. Olanzapine was chosen as it possesses all these characteristics: it has a high melting point (197 °C); it is stable up to this temperature; it is poorly soluble in water; and it is administered at dosages ranging from 5 to 20 mg. Moreover, the melting method to prepare a solid dispersion is preferable to the solvent one, as olanzapine forms solvates with most solvents [2].

Olanzapine was previously formulated as a solid dispersion in pre-gelatinized starch and sodium glycollate, obtaining a marked increase in aqueous solubility, dissolution rate and an improved drug release profile with respect to the pure drug [3]. The drug was also associated with hydrophilic polymers with the aim of improving the dissolution parameters in different formulations, such as freeze-dried tablets with micronized gelatine [4], controlled release tablets in the presence of methocel and ethocel [5], long acting microspheres formed by different polylactic/polyglycolic acid (PLGA) co-polymers [6], or in the form of inclusion complexes with cyclodextrins [7,8].

No study could be found in the recent literature concerning the association olanzapine/polyethylene (or prolypropylene) oxide. Polyethylene/polypropylene oxide (PEO/PPO) co-polymers were shown to be more versatile than the corresponding single polymers in promoting the dissolution rate and also solubility of poorly soluble pharmaceutical actives [9,10]. These co-polymers are commercially available with different trade names (Pluronics, Poloxamers, Lutrols…) and are identified by a number related to the molecular weight and composition. The most common polymers of this class are designed as di-functional tri-block copolymers that contain a central block of relatively hydrophobic PPO, surrounded on both sides by the blocks of the relatively hydrophilic PEO. Due to the possibility to combine blocks of different length and molecular weight, the properties of the resulting polymers vary in a wide range in terms of molecular weight, different appearance (liquid, paste, and solid) and variations in the hydrophilic-lipophilic balance (HLB) of the individual compounds. Above a molecular weight of 3000, the materials are generally waxy, white granules of a free-flowing nature and are practically odorless and tasteless, widely used in pharmaceutical formulations as wetting and emulsifier agents, particularly suitable for preparing solid dispersions to improve solubility and dissolution parameters for low soluble actives in solid oral forms. Lutrol^®^ F68 and Lutrol^®^ F127 were selected in this paper as low meltable carriers and also for their surfactant activity and ability to form polymer micelles with solubilizing properties towards hydrophobic drugs [11,12].

Gelucires represent a class of materials that could contain mixtures of mono-, di- and tri-glycerides of fatty acids of different chain lengths that may contain also and/or mono- and di-esters of the same or different fatty acids of PEO of different chain lengths; they are characterized by a range of melting points and HLB values: they have proved suitable to prepare formulations of disperse phase systems, with enhanced solubility and dissolution rate, and modified release. Gelucire^®^ 44/14 is a non-ionic surface-active excipient of the Gelucire class, largely used to produce immediate-release from solid dosage forms for poorly soluble drugs. In particular, it is mainly composed of mono-and di-laurate (C12) PEO esters and a small glyceryl laurate fraction and free PEO. It is a semi-solid waxy (*m.p.* 44 °C), amphiphilic (HLB 14) and safe material for oral formulation: it allows rapid release and has been widely studied with a variety of drugs and formulations in order to enhance the dissolution rate [13,14]. In the present work, Gelucire 44/14 was considered as an additional carrier to improve the release and, possibly, the solubility of olanzapine in binary carriers with Lutrol^®^ F68 and F127: its formulation as a mixture enables negligible variations related to polymorph transformation, preventing the phenomena expected from a single component carrier, though maintaining a high HLB value [15].

An attempt was made to insert Transcutol^®^, as a liquid component, into a Lutrols/Transcutol ternary carrier both to increase solubility of the drug and stability of the solid dispersion.

Embedding olanzapine in mono, binary or ternary systems should guarantee a hydrophilic environment, enabling its easy release and dissolution and, at the same time, should prevent amorphization or crystallization processes of the active agent, sources of the changes that occur with aging and points of weakness when considering solid dispersions for practical and commercial purposes.

## 2. Methods

### 2.1. Materials

Olanzapine was a gift of pharmaceutical grade (Montefarmaco OTC, Bollate-Milan, Italy): the sample was crystallized for purification by cooling an anhydrous ethyl acetate solution that gives the stable form. Its thermogram fits that of a commercial sample (*m.p.* 197 °C). Lutrol F68^®^ and F127^®^ were commercial samples, purchased from BASF AG (Ludwigshafen, Germany); and Gelucire 44/14^®^ (lauroyl macrogolglycerides (polyoxylglycerides: *m.p.* 50 °C; HLB 14) and Transcutol^®^ (diethylene glycol monoethyl ether) were obtained as gift samples from Gattefosse (Saint-Priest, France), of the highest purity available.

### 2.2. Preparation of the Physical Mixtures

Physical mixtures were prepared by mixing olanzapine with the carrier in the form of binary and ternary mixtures with Lutrols and Gelucire, according to the ratios listed in Table 1 in order to obtain 11 formulations. For systems 6, and 9–11, the single carriers were mixed in the weight ratios shown in Table 1 and then were fused together and cooled. After solidification, the mass was milled and used as a carrier to prepare the single physical mixtures by addition of olanzapine.

### 2.3. Preparation of the Solid Dispersions

A total of 0.5 g of each physical mixture was melted in a porcelain plate with a gradual increasing of the temperature up to a value necessary for the complete melting. At about 10 °C above the melting temperature of the main carrier (or of the mixture), the systems were transparent and limpid. The systems thus prepared were placed in a freezer at −20 °C for 24 h and then crushed, milled, and sieved at room temperature; powders with 100 < *x* < 200 μm size were used for further tests.

### 2.4. X-ray Diffraction (XRD)

Some of the systems in the form of powders were analyzed by X-ray diffraction technique using a Philips PW 3719 diffractometer controlled by a computer. Experimental conditions: Cu Ka radiation (λ = 1.78896 Å); 40 kV and 30 mA. Scanning interval: 0–50 °2θ; Time per step: 1 s; Graphite monochromator on the diffracted beam.

### 2.5. Scanning Electron Microscopy (SEM)

SEM images were taken by a Philips XL30 microscope: samples were previously sputter-coated with a gold layer in order to make them conductive.

### 2.6. Differential Scanning Calorimetry (DSC)

Thermograms were obtained with Mettler equipment (FP 80HT control unit, FP 85TA cell furnace and FP 89 control software). Samples of about 10 mg were accurately weighed and analyzed in pierced Al crucibles in the range of 40–300 °C, at a heating rate of 10 °C min^−1^. For comparison of thermal behavior of the systems, the temperature of the peak was considered. Strict respect of the heating and cooling times were parameters observed to ensure reproducibility to the results in fresh and aged systems.

### 2.7. Thermomicroscopy (HSM)

Hot-stage microscopy was carried out by means of a Mettler FP 82HT hot plate, coupled to an Olympus BH-2 optical microscope, equipped with a photographic recorder (Olympus C-35AD-4). A Mettler FP 80HT control unit was used to control the heating rate of the hot plate in the range of 25–300 °C, with a scan rate of 10 °C min^−1^ or lower in the proximity of the most interesting thermal events.

### 2.8. Dissolution Rate Studies

Dissolution profiles were obtained using a USP XXIX paddle method (Turu-Grau mod. D-6 apparatus), evaluating 10 mg of pure olanzapine—as a working concentration—or equivalent amounts of each sample. The dissolution medium was 1000 mL of twice-distilled water at 37 °C at 50 rpm; an amount of solid dispersion equivalent to 10 mg of olanzapine was added to the dissolution flask. Withdrawals were obtained at preset times and the drug concentration was measured spectrophotometrically at 276 nm. Studies were conducted for a period of 1 h with the above fixed parameters in triplicate. The average amount of olanzapine released was then calculated from the recorded values and reported in percent terms.

## 3. Results and Discussion

### 3.1. Solid Dispersions (Systems 1–11)

Olanzapine, a new generation atypical antipsychotic belonging to the class of tienobenzodiazepines, appears as a pale yellow crystalline powder and is a highly melting molecule, well organized in the solid state, where units are compactly bound together by a network of H-bonds. This drug crystallizes in different solid anhydrous polymorphs, hydrates (with varying degrees of hydration) and a large number of solvates [2].

Many physical and chemical parameters of this molecule have been determined that depict a hydrophobic, poorly soluble, and poorly polar molecule. Moreover, the organization of the solid state demonstrates high efficiency, since it remains unchanged despite the inclusion of a number of foreign molecules in the formation of hydrates and solvates. Olanzapine was chosen as a model drug in this paper: in fact, it is usually administered at a very low dosage (5–20 mg): its association with an inert carrier, that causes a weight increase of the final formulation, constitutes an irrelevant problem compared to the important improvement of the release. In addition, olanzapine is a high melting compound (197 °C) and in this work the unsolvated form 1 was employed [2], stable at high temperatures, as it melts, unchanged at 197 °C.

The carriers, alone (systems 1–5 and 8) or in binary (systems 6, 7, 9, and 10) and ternary (system 11) mixtures, were chosen for the high HLB, which represents an appropriate parameter to provide for an immediate release of the embedded drug. The concentration 10% *w*/*w* of olanzapine was examined, and the effect of its concentration was also evaluated (systems 1–7) (Table 1) for possible practical applications.

**Table 1:** Percent composition of solid dispersions.

Systems	1	2	3	4	5	6	7	8	9	10	11
Components (% *w*/*w*)											
Olanzapine	15	10	5	15	5	15	10	10	10	10	10
Lutrol^®^ F68	85	90	95			42.5	45		45		30
Lutrol^®^ F127				85	95	42.5	45			45	30
Gelucire^®^ 44/14								90	45	45	
Transcutol^®^											30

### 3.2. DSC Thermograms of the Mixed Carriers

All the carriers considered here are low melting materials, suitable for the melting method of the solid dispersion preparation that avoids the use of organic solvents and the problems accompanying their use in this process [16].

The thermogram baselines show that carriers and drug are thermally stable before and after the melting, making the melting method to prepare the solid dispersions both attractive and safe. The preparation of the molten mixture that was carried out at about 80 °C could be continued for a long time, or even at higher temperatures up to complete dissolution of the drug into the molten carrier, without any problem of loss or change of the drug.

**Figure 1:**
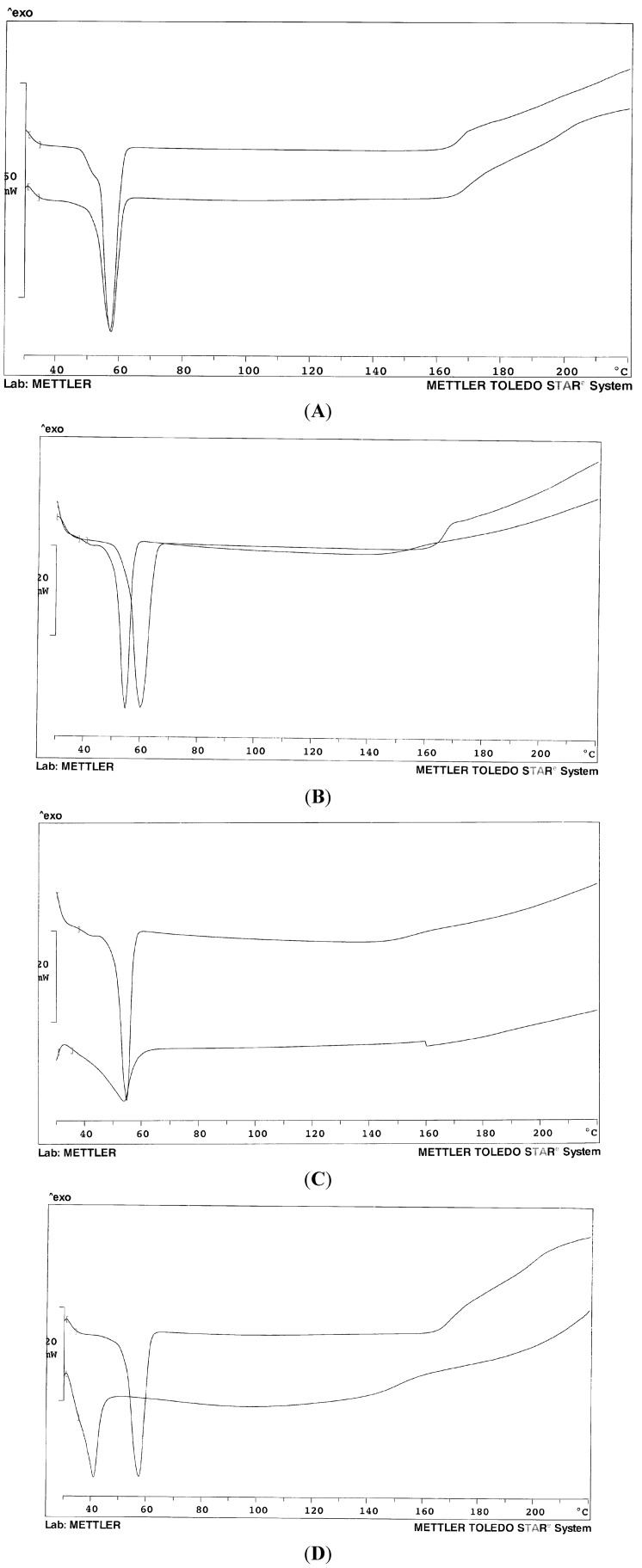
Thermogram profiles of the mixed carriers in comparison with a single carrier. (**A**) Mixed Lutrol^®^ F68 and F127 (50% *w*/*w*) (below) and Lutrol^®^ F68 (above) for comparison; (**B**) Mixed Lutrol^®^ F127/Gelucire^®^ 44/14 (50% *w*/*w*) (**left**) and Lutrol^®^ F127 (**right**) for comparison; (**C**) Mixed Lutrol^®^ F127/Gelucire^®^ 44/14 (50% *w*/*w*) (**above**) and Gelucire^®^ 44/14 (**below**) for comparison; (**D**) Mixed Lutrol^®^ F68, Lutrol^®^ F127 and Transcutol (33.3% *w*/*w*) (**below**); Mixed Lutrol^®^ F68 and F127 (50% *w*/*w*) (**above**) for comparison.

Thermal analysis of the systems prepared with modified conditions did not reveal any difference or degradation. Figure 1 shows the thermogram profiles of the mixed carriers in comparison with the single component: it can be appreciated that the resulting endotherm is often somewhat broader and displays a lower temperature peak than that of the single component and this argues in favor of a mutual dissolution. Due to similar chemical composition and comparable nature, the mixtures of the two Lutrols do not display thermograms different with respect to the single polymers (Figure 1A).

While the melting peak of Gelucire^®^ 44/14 is very broad and asymmetric, due to the complex composition of this material, in the presence of the Lutrol, the endotherm of the mixture is more regular (Figure 1C) and the baseline stable up to high temperatures; the system could be thus described as Gelucire dissolved into the polymer (Figure 1B), as the presence of Gelucire decreases the melting temperature of the Lutrol. Finally, Figure 1D shows the dramatic lowering of the melting point of the system in the presence of a liquid component that modifies the whole system into a semi-solid mass.

### 3.3. DSC Thermograms of the Solid Dispersions

**Figure 2:**
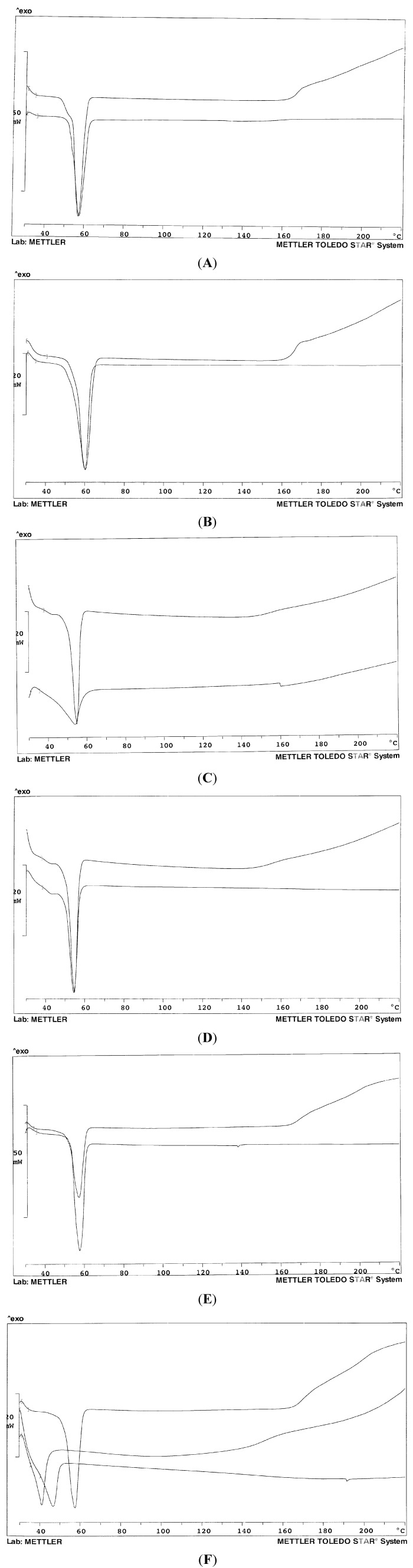
Thermogram profiles of: (**A**) Lutrol^®^ F68 (**above**) and system 3 (**below**); (**B**) Lutrol^®^ F127 (**above**) and system 5 (**below**); (**C**) System 8 (**above**) and Gelucire^®^ 44/14 (**below**); (**D**) Lutrol^®^ F68 and Lutrol^®^ F127 mixture 50% *w*/*w* (**above**) and system 7 (**below**); (**E**) Lutrol^®^ F127 and Gelucire^®^ 44/14 mixture 50% *w*/*w* (**above**) and system 10; (**F**) Lutrol^®^ F68 and Lutrol^®^ F127 mixture 50% *w*/*w* (**above**); Lutrol^®^ F68, Lutrol^®^ F127 and Transcutol^®^ mixture 33.3% *w*/*w* (**middle**); system 11 (**below**). The percent concerns the composition of the carriers.

Figure 2A–F show the thermogram profiles of some systems of Table 1 in comparison with that of the carrier (mixed or single). The pure carriers proved to be rather stable up to about 160 °C: the baseline then suggests decomposition; on the contrary, when formulated as a solid dispersion with olanzapine, it appears that the drug somehow stabilizes the systems, at least up to 200 °C, since the baseline does not show any deviation. The thermograms in Figure 2A,B show only the melting endotherm of the solid dispersion for systems 3 and 5 containing only Lutrol^®^ F68 and F127, respectively: no peak is evident related to the melting of the drug. Melting endotherms of the pure carrier and of the dispersion perfectly overlap in both cases, despite the presence of the drug: this fact suggests a very low concentration of dissolved olanzapine in the solidified carrier at room temperature. In other words, at room temperature, Lutrols are poor solvents for olanzapine that could dissolve into the molten Lutrol at the temperature of the preparation of the solid dispersion and that massively precipitates when the system has cooled: this fact was confirmed by thermomicroscopy (see below). The absence of the melting peak of olanzapine (at 197 °C) indicates its dissolution into the molten carrier at increasing temperatures during the recording of the thermogram. No differences were observed on changing the content of olanzapine in systems 1–5 or 6 and 7 (thermograms not shown): this could indicate that all the systems studied contain the drug at a concentration higher than its saturation in the carriers. The same was observed when the two Lutrols were mixed together (systems 6 and 7) (Figure 2C): the two Lutrols do not carry out any synergism towards dissolution of olanzapine at room temperature; they differ for the different length of the three chains that build the block co-polymer, which does not affect the result of the dispersion in the solid state: in fact the melting points are close together. The situation does not change when the two Lutrols are mixed together (system 7, Figure 2D).

On the contrary, Gelucire^®^ 44/14 appeared to be a good solvent for olanzapine both at high and low temperatures: the thermogram shows a notable difference between the pure carrier and system 8 (Figure 2C) that contains only Gelucire^®^ 44/14 as a carrier. However, Gelucire-containing systems are difficult to handle: they are pasty and semi-solid, cannot be milled or sieved, and the particles do not show defined borders or shape. When Lutrol and Gelucire are mixed together at the same weight ratio, there is no difference between the carriers and system 9 (and 10) in terms of peak temperature, which is a sign of the absence of dissolved drug within the systems (Figure 2E). Interestingly, Gelucire^®^ 44/14 has a broad and asymmetric thermogram profile, as a consequence of its composition: however, when used as a solid dispersion with olanzapine, alone or with Lutrol, the melting endotherm appears more symmetric and the baseline stable up to high temperatures.

Figure 2F shows the thermogram of system 11, where a 30% *w*/*w* of Transcutol^®^ was added to promote solubility of the drug, even at low temperatures: the system was difficult to handle, due to its low melting temperature and sticky nature; it shows a broad melting endotherm centred at 46 °C, different from those of the binary mixture of the two Lutrols or the ternary mixture at 33.3% *w*/*w* of each component. Due to the presence of a liquid component, system 11 was difficult to solidify: a small endotherm suggests the presence, though poorly appreciated, of crystalline olanzapine.

### 3.4. Solubility Parameters

Carriers for solid dispersions behave as solvents for the drug in the molten state; solubility at high temperatures is an important parameter that guarantees dispersion of the drug at molecular level, which is a prelude to a notable help for particle size reduction and promotion to dissolution. In fact, when the molten system becomes colder, the “solvent” rapidly solidifies and, due to increased viscosity, the crystallization process concerning the dissolved drug is slowed down: nucleation of crystalline germs is difficult, and diffusion, necessary to increase and complete the crystal lattice of the drug particles, is seriously limited. The drug remains dispersed inside the mass of the solid carrier, as it was in the molten phase, which is practically in an amorphous form. These facts encompass the positive and negative aspects of solid dispersions. An amorphous state is very favorable for an increased dissolution rate of the dispersed drug; but also for a potential pathway towards crystallization that should negatively affect the performance of the system. As a consequence, the solubility of the drug in the solid carrier is an invaluable parameter to predict the stability of solid dispersions with aging. A high solubility of the drug in the carrier at high temperatures, which is maintained also at room temperature, can generate a metastable situation, which evolves with aging towards crystallization or the increase in the size of the precipitated particles. This fact, not always predictable, is one of the phenomena associated with the change of solid dispersions with time, which alters their performance on release and inhibits large-scale commercial development of these systems. Therefore the best situation that can be encountered in a solid dispersion, for a relatively guaranteed stability, should be the one that contains the drug as precipitated and crystalline particles or as a dissolved phase inside a carrier of sluggish crystallization.

The use of the solubility parameter δ, as a tool to predict the solubility of a compound in solvents, but also in molten systems, is a great aid for the technologist. Simply, a large difference between the values of δ between the solute and the solvent is a sign of lack of mutual affinity and hence solubility; such information must, however, be considered with caution, because the systems considered (especially those in the molten state) are often far from ideal. However, it does allow a pre-selection of the most suitable solute/solvent pairs to fit the desired purposes.

A δ value of 19 (MPa)^1/2^ can be found for Lutrol^®^ F68 [17], and a value 21.09 (MPa)^1/2^ for Gelucire [18], that in the present systems could behave as solvents (in the molten state) for olanzapine. The solubility parameter δ cannot be found in the recent literature for olanzapine, but it could be simply determined using the method of the solubility peak. The problem of the formation of solvate with most solvents limits the choice of solvents, enabling measurements of solubility for unsolvated olanzapine, that is the form used in the present work: olanzapine was found to crystallize unsolvated from acetone, ethyl acetate, toluene, and ethyl ether [16], whose solubility parameters are 9.77, 9.10, 8.91, and 7.62 (MPa)^1/2^ respectively [19]. As these solvents display very close δ values, it could be estimated that the δ value for olanzapine, when determined by the solubility peak method, should fall in the range 9.77–7.62 (MPa)^1/2^, which represents a fairly low value. Therefore, the Δδ between Lutrol (and Gelucire) and olanzapine makes it possible to predict a low solubility of olanzapine in Lutrols or Gelucire: as a consequence, the amount of the dissolved drug inside the solid dispersion is expected to be low at room temperature, without preventing good solubility at higher temperatures (as was observed). As this aspect should limit the effect of aging in terms of crystallization of the drug, it follows that the preliminary choice of Lutrols and Gelucire as carriers for solid dispersion of olanzapine is suitable to ensure stability to the dispersed systems. In most of the present cases (systems 1–7), the olanzapine/Lutrol pair forms systems where the drug is expected to be poorly dissolved in the carrier and, therefore, better considered at room temperature as a real dispersion in the solid state. The same is expected in the presence of Gelucire^®^ 44/14: however, due to the heterogeneous nature of Gelucire, a difficult process of crystallization of the olanzapine dissolved at high temperature is expected.

### 3.5. X-ray Diffractograms

**Figure 3:**
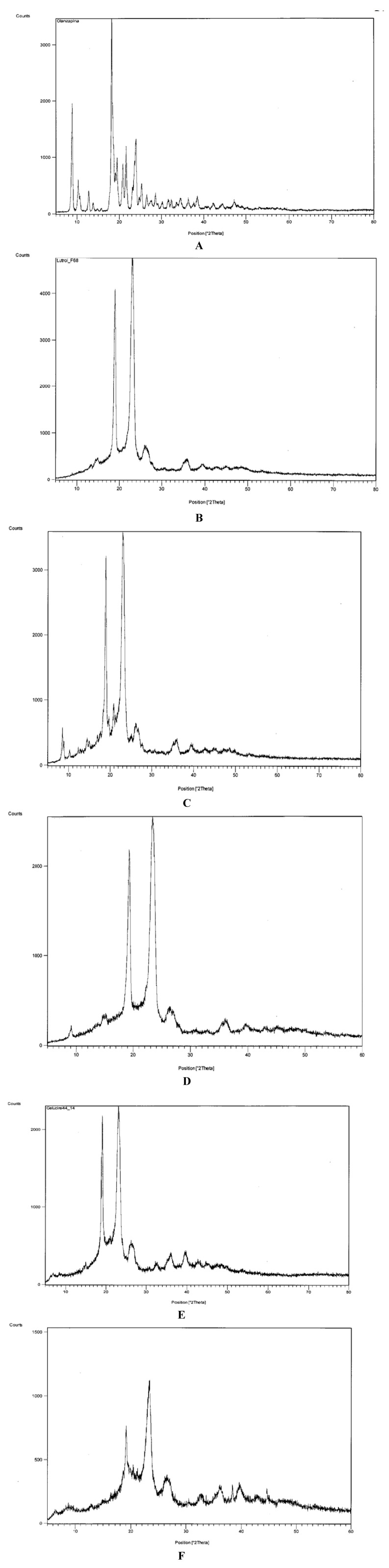
Diffractograms of: (**A**) pure olanzapine; (**B**) pure Lutrol^®^ F68; (**C**) system 3; (**D**) system 5; (**E**) Gelucire^®^ 44/14; (**F**) system 8.

Figure 3 shows diffractograms that confirm the presence of crystalline olanzapine inside some of the systems examined.

Figure 3A shows the diffractogram of olanzapine: two distinctive groups of peaks of the compound can be seen: those in the range 8–15 °2θ, particularly the peaks at 8.88 and 10.29 °2θ, are relatively intense and are useful to identify olanzapine in the solid dispersions.

In fact, both Lutrols (and Gelucire^®^ 44/14) have intense peaks at about 19 and 23 °2θ (Figure 3B,E) that overlap those of olanzapine present in the same range and therefore cannot be useful for its identification (Figure 3A). Figure 3C,D show diffractograms of systems 3 and 5 containing olanzapine at 5% *w*/*w* in Lutrol^®^ F58 and F127 respectively: a partially split peak concerning olanzapine can be seen inside the circle, more intense in system 3 (Figure 3C), indicating its crystallinity (confirming the idea derived from the solubility parameters and the observation with the thermomicroscope—see below). The intensity of the peaks is obviously low, as a consequence of the low concentration of olanzapine in the systems. On the contrary, Figure 3F, related to system 8, where olanzapine was found dissolved inside Gelucire^®^ 44/14, no peak could be found associated with crystalline olanzapine, indicating its dissolution in the system.

### 3.6. Thermomicroscopy (HSM)

Thermomicroscopy confirms what is suggested by DSC: after the melting of the carrier, the surface of the molten vesicles appears completely occupied by olanzapine particles, recognizable by the yellow color, and reduced size: this was observed in all the cases containing Lutrols alone or in mixture (systems 1–7). At increasing temperatures, the particles progressively dissolve into the molten carrier. This means that, at the time of preparation of the solid dispersion, the drug was dissolved in the molten carrier; on cooling numerous crystalline germs of the drug emerge from the molten phase that do not have time to grow before the solidification of the carrier. In this respect the solubilizing capacity of the different carriers can be roughly estimated by the temperatures of the complete dissolution.

**Figure 4:**
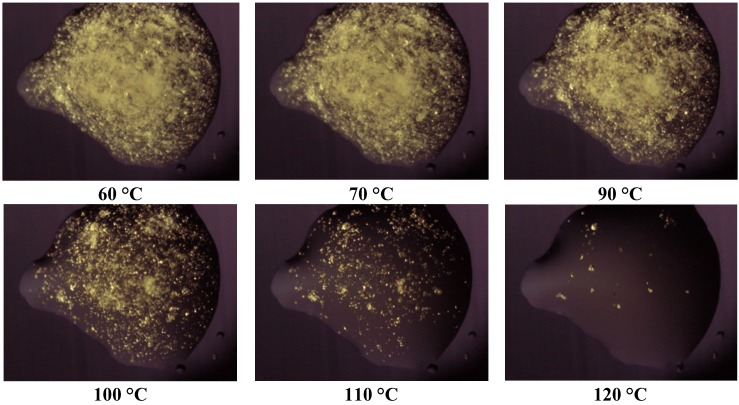
Photos of the system 1 taken at thermomicroscope at increasing temperatures.

The photos of Figure 4 prove that Lutrol^®^ F68 is a poor solvent at low temperatures, but a good one at high temperatures (system 1); the same could be reported for the systems containing Lutrol^®^ F127. It emerges that the solubility of olanzapine in both Lutrols or their mixtures, examined here is lower than the lowest concentration used: that is in all systems 1–7 olanzapine concentration is above the saturation. This means that the amount of the drug dissolved is very low and, as suggested by the same melting point of the carrier and the solid dispersion, not enough to generate a decrease of the melting point of the system (cryoscopic effect): this aspect argues in favor of the stability of the systems containing Lutrol.

**Figure 5:**
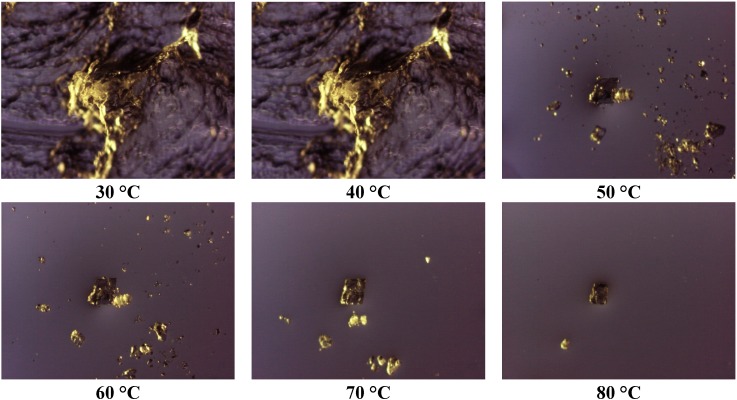
Photos taken at thermomicroscope of system 8 at increasing temperatures.

**Figure 6:**
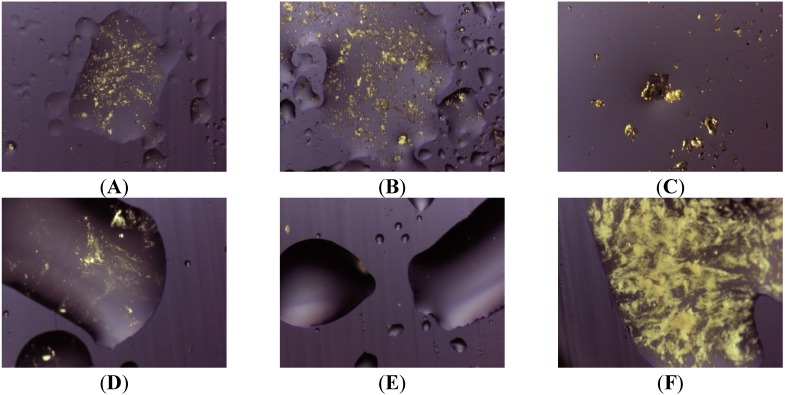
Photos taken at thermomicroscope after melting of the carrier (**A**) System 8 freshly prepared; (**B**) System 8 aged one year; (**C**) System 8 at 10% *w*/*w* olanzapine; (**D**) System 8 at 5% *w*/*w* olanzapine; (**E**) System 8 at 2.5% *w*/*w* olanzapine; (**F**) System 11 prepared by heating up to 100 °C.

The presence of Gelucire^®^ 44/14 apparently increases the solubility of olanzapine: the photos (Figure 5, system 8) show that only a very small number of particles of undissolved olanzapine are present inside the molten mass of the carrier, confirming that this system is also saturated in olanzapine. This situation does not change when solid dispersions aged more than one year are examined (see Figure 6). These views appear to contradict what is indicated by the solubility parameters reported for Gelucire that allowed the hypothesis of a low solubility of olanzapine in the Gelucire. However, this could be the result of a metastable situation (dissolution at high temperature that did not evolve to crystallization at low temperature) rather than a real solvent ability of Gelucire towards olanzapine (see below). The same system, subjected to ultrasound discharge, revealed the presence of crystallized olanzapine, suggesting that crystallization could be achieved in this metastable system by applying suitable processes.

In fact, the nature of Gelucires, formulated as a complex mixture, enables stability, as the composition makes processes such as crystallization or polymorph transition with aging difficult, like those described for single triglycerides. These phenomena are usually accompanied by a change from an apparent homogeneous system towards a heterogeneous system with consequent de-mixing or exclusion of the foreign material, such as a solute in a solid dispersion, outside the re-crystallized matrix. A higher solvent ability of Gelucire, that is a mixture of mono-, di-, and tri-glycerides (and esters of short chain PEO), than that of a single triglyceride can, therefore, be expected toward olanzapine at high temperatures; but also high stability of the dissolved material at room temperature, only related to a hindrance to crystallization. The presence of dissolved drug usually plays both positive and negative roles in solid dispersions.

The dissolved drug in a hydrophilic matrix represents the optimum for rapid dissolution; however, in the presence of dissolved material, in a metastable state, potential crystallization into particles of increasing size could be expected, *i.e.*, changes of the formulation that could affect its behavior in solution or stability in the solid state. Thermal analysis of aged samples confirms stability, as it shows that these systems do not differ from the “young” ones (Figure 6A,B). Pure Gelucire^®^ 44/14 could therefore be suitable to prepare a homogeneous system: however, in the presence of Lutrols (systems 9 and 10) the solubility of olanzapine again decreases, though its dissolution in the molten carrier at increasing temperatures is more rapid than in systems 1–7. Figure 6C–E show the effects of the concentration on system 8: even though only a few particle of olanzapine can be found undissolved for 10% and 5% *w*/*w* concentrations; a complete dissolution of the drug in Gelucire^®^ 44/14 could be observed for the lowest concentration (Figure 6E).

The addition of Transcutol^®^, as an aid to improve solubility of olanzapine in system 11, was unsuccessful: this solvent actually has the formula 2-(2-Ethoxyethoxy)ethanol that somehow recalls the structure of a Lutrol and, accordingly, does not behave as a solvent for olanzapine. Figure 6F clearly shows the presence of precipitated particles of drug, despite the preliminary heating up to 100 °C of the molten carrier to obtain a complete dissolution at the time of preparation of system 11. All these facts suggest that the presence of Transcutol^®^ does not offer any advantage to solid dispersions.

### 3.7. Dissolution Tests

The thermal analysis showed that systems 1–11 can be divided into two classes: those where olanzapine is present as crystalline particles and those where the drug is dissolved inside the carrier. It is expected that this difference plays a role in the release of the drug.

**Figure 7:**
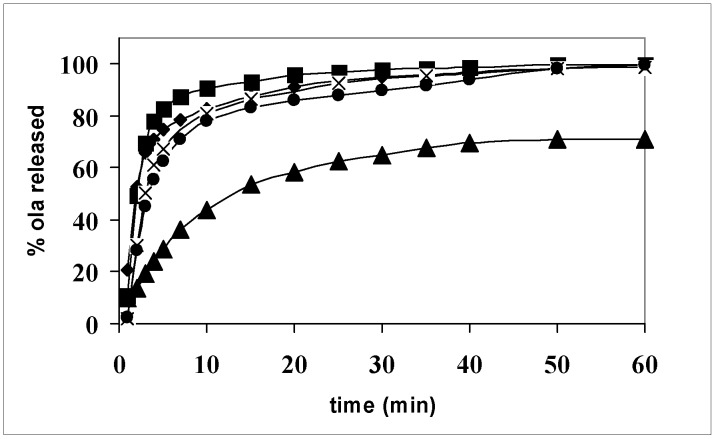
Release profiles of olanzapine from: ▲ powder (size fraction in the range 100–200 μm); ● system 8; **×** system 2; ■ system 5.

Figure 7 shows the release profiles of olanzapine from a single carrier (systems 2, 5, and 8) and the action of the hydrophilic carriers to promote olanzapine dissolution with respect to the pure drug is evident: the release is completed in less than 1 h, while pure olanzapine dissolves 70% in the same time. Differences among the carriers could be detected after 10 min dissolution: system 8 contains the drug, practically dissolved, but Gelucire^®^ 44/14 appears a less efficient promoter than the Lutrols, where, on the contrary, the drug is in the form of small size particles, as shown by the thermomicroscopy (see Figure 4 and Figure 5). It therefore appears that, provided a drug is embedded in a strongly hydrophilic carrier, its physical form (dissolved or precipitated) has a limited relevance to its dissolution rate. The small differences of the dissolution profiles can be probably attributed, not to the differences in the solid state, but to the different behavior of the carrier in aqueous solution. Lutrols, in fact, when dispersed in aqueous solutions at low concentrations, exist as monomolecular micelles, where the PPO block forms the central hydrophobic core able to solubilize hydrophobic substances, while water solubility of the whole chain is due to the PEO side blocks. Therefore, in Lutrol-containing systems, together with the high HLB that affects wettability, micellar solubilization in the dissolution medium operates to further improve the release rate of olanzapine and can explain the small differences in terms of the release rate observed in Figure 7. In addition the two Lutrols differ in molecular weight and the length of the chains of the three blocks. In particular, Lutrol^®^ F127 has its central chain formed by 56 PPO units, double the value with respect to Lutrol^®^ F68, which means a larger-size core making it possible to promote a better solubilization of the drug and thus a better release rate from the solid dispersion. The release profiles of the aged systems (not shown) do not change after one year, demonstrating the stability of the formulation.

## 4. Conclusions

Drug and carriers were selected for the preparation of the solid dispersion according to their different solubility parameters that could guarantee stability with aging.

Olanzapine is a model drug suitable to be formulated as a solid dispersion since its very low dosage is not affected by the weight increase of the formulation related to the presence of the carrier. Carriers and drug are thermally stable and not affected by heating of the melting method to prepare the solid dispersion.

The Lutrols and Gelucire examined in this paper (alone or in mixtures) guarantee stability of the olanzapine-containing systems with different mechanisms. Stability of the solid dispersions is assured by the low solubility of olanzapine in the Lutrols: the drug dissolves at high temperatures and precipitates on cooling in the form of crystalline particles of reduced size. The presence of Gelucire in the carrier enables dissolution of olanzapine in the solid dispersion at room temperature, though in a metastable state.

The systems prepared with the proposed hydrophilic carriers do not change performance to the release over time. Both types of carriers promote a quick release of olanzapine from the solid dispersion. The ability of Lutrols to form polymer micelles in aqueous solution make all the formulations that contain them very similar to each other: all of them display the same accelerated release of the drug.
